# Effect of azithromycin in combination with simvastatin in the treatment of chronic obstructive pulmonary disease complicated by pulmonary arterial hypertension

**DOI:** 10.12669/pjms.332.11717

**Published:** 2017

**Authors:** Peidong Wang, Jie Yang, Yanwei Yang, Zhixin Ding

**Affiliations:** 1 Peidong Wang, Department of Pulmonary Disease, Zhengzhou Hospital of Traditional Chinese Medicine, Zhengzhou, 450007, China; 2 Jie Yang, Department of Pulmonary Disease, Zhengzhou Hospital of Traditional Chinese Medicine, Zhengzhou, 450007, China; 3 Yanwei Yang, Department of Pulmonary Disease, Zhengzhou Hospital of Traditional Chinese Medicine, Zhengzhou, 450007, China; 4 Zhixin Ding, Intensive Care Unit, Zhengzhou Hospital of Traditional Chinese Medicine, Zhengzhou, 450007, China

**Keywords:** Azithromycin, COPD, Simvastatin

## Abstract

**Objective:**

To evaluate the effect of azithromycin in combination with simvastatin in the treatment of chronic obstructive pulmonary disease (COPD) complicated by pulmonary arterial hypertension.

**Methods:**

Eighty-six patients who developed COPD and pulmonary arterial hypertension and received treatment from August 2013 to October 2014 were selected and randomly divided into an observation group and a control group using random number table, 43 in each group. Patients in the control group were orally administrated 20 mg of simvastatin, once a day. Patients in the observation group took 0.25g of azithromycin enteric-coated tablets, once a day, besides simvastatin. The treatment course of both groups was six months. Blood gas analysis indexes, forced expiratory volume in first second (FEV_1_), six minutes walking distance, dyspnea grade and blood lipid parameter were recorded and compared between the two groups.

**Results:**

Arterial partial pressure of oxygen (PaO_2_) and arterial partial pressure of carbon dioxide (PaCO_2_) of the observation group were (68.13±3.03) mmHg and (45.08±2.27) mmHg after treatment, respectively. In the control group, the values were (60.01±4.72) mmHg and (38.93±1.61) mmHg, respectively. The improvement amplitude of the observation group was superior to that of the control group (*P*<0.05). FEV_1_, forced vital capacity (FVC) and 6-minutes walking distance of the observation group were (1.08±0.11) L, (2.1±0.2) L and (380.34 ± 31.28) m respectively after treatment, superior to the control group ((0.93±0.09) L, (1.7±0.1) L) and (302.79±29.74) m, and the difference had statistical significance (*P*<0.05). The levels of peripheral systolic blood pressure (PSBP) and peripheral diastolic blood pressure (PDBP) of patients in the observation group were both lower than those of the control group, and the difference had statistical significance (*P*<0.05).

**Conclusion:**

Azithromycin in combination with simvastatin has definite effect in the treatment of COPD in combination with pulmonary arterial hypertension as it can significantly relieve ventilation disturbance, improve lung function, and decrease pulmonary arterial pressure. Hence it is worth clinical promotion.

## INTRODUCTION

Chronic obstructive pulmonary disease (COPD), a commonly seen disease in respiratory system, can induce ventilation/perfusion defects and massive loss of alveolar capillary network, leading to anoxia, carbon dioxide retention and secondary pulmonary hypertension. COPD has a long disease course. If not treated timely, it may develop to pulmonary heart disease, which can severely affect the quality of life of patients.^1^,^2^ COPD induced pulmonary arterial hypertension, a systemic inflammatory response, affects multiple organs of patients by means of inflammation and oxidative stress injury.^3^ Peinado VI et al. found that,^4^ timely and effective control on pulmonary artery pressure could significantly relieve symptoms and improve prognosis.

Currently, main treatment methods for COPD in combination with pulmonary arterial hypertension focus on inducement such as infection.^5^ A study found that,^6^ macrolide antibiotics including azithromycin and erythromycin could resist bacteria and inflammation and lower the risks of acute aggravation of COPD. Statins can selectively act on 3-hydroxy-3-méthylglutaryl-coenzyme A (HMG-CoA) reductase, significantly lower the synthesis of cholesterol, regulate blood lipid, and independently protect blood vessels. Statins have been extensively used in cardiovascular medicine in the past, and it is found effective in lowering pulmonary arterial hypertension.^7^ Zhao Xianming et al. found that,^8^ statins could improve the ventilation function of COPD patients and also could resist inflammation and vascular reconstruction, suggesting an enormous role in the clinical treatment of COPD associated pulmonary arterial hypertension. Cheello et al. found that,^9^ simvastatin could improve the inflammatory effect of glucocorticoids, delay the decline of lung function of patients with COPD, and reduce acute attack frequency. This study was carried out to investigate the clinical effect of azithromycin in combination with simvastatin in the treatment of COPD in combination with pulmonary arterial hypertension.

## METHODS

Eighty-six patients who were diagnosed as COPD in combination with pulmonary arterial hypertension and treated in the Zhengzhou TCM Hospital from August 2013 to October 2014 were selected. This study had been approved by the ethics committee of the Zhengzhou TCM Hospital. The patients had signed informed consent.

Patients whose mean arterial pressure was detected as not less than 25 mmHg by right cardiac catheterization in a quiescent condition or as no less than 30 mm Hg in a motion state and patients who had not suffered from acute attack of COPD or acute lung infection were included.

Patients who had severe cardiac, hepatic and liver function abnormality, pulmonary thromboembolism, allergic rhinitis, asthma or primary pulmonary hypertension or were allergic to the drugs used in the study were excluded.

### Drugs and instruments

Simvastatin (specification: 40 mg/pill; batch no.: 090601; Hangzhou Merck Sharp & Dohme Pharmaceutical Co., Ltd., China), azithromycin enteric-coated tablets (Specification: 0.25 g/pill; Batch no.: 1011101; Zhejiang Dade Pharmaceutical Group, China), SN670846 pulmonary function detector (Jaeger Company, Germany), CobasB12 blood gas analyzer (Roche Group, Switzerland) and Modalar DDP RASpro EC2 fully automatic biochemical analyzer (Roche Group, Switzerland) were used.

### Methods

Patients in both groups received conventional treatment including continuous low-flow oxygen inhalation, rest in bed, monitoring of vital signs, electrolyte and acid-base balance as well as symptomatic treatment including cough relief and antiasthma using aminophylline and β2 receptor agonist. Patients in the control group orally took simvastatin tablets at night, 20 mg once. Patients in the observation group took azithromycin tablets, 0.25g once, once a day, besides simvastatin tablets. The treatment lasted for six months in both groups.

### Observation index

Arterial blood was collected from each patient before and after treatment. Arterial oxygen pressure (PaO_2_), arterial partial pressure of carbon dioxide (PaCO_2_) and pH value were detected using a CobasB12 blood gas analyzer. Forced expiratory volume in one second (FEV_1_), forced vital capacity (FVC), six minutes walking distance and pulmonary arterial pressure were recorded after treatment.

### Statistical analysis

Data were statistically analyzed by SPSS ver. 20.0. Measurement data were expressed as mean±standard deviation (SD). The comparison between two groups was performed using independent sample t test. The comparison of data within groups before and after treatment was performed using paired t test. Enumeration data were compared using Chi-square test. Difference was considered statistically significant if *P*<0.05.

## RESULTS

In the control group (43 cases), there were 27 males and 16 females, with an average age of (72.43±8.28) years old (61~83 years old) and an average disease course of (10.91±5.02) years; there were 11 cases of cardiac functional grade II, 23 cases of grade III and 9 cases of grade IV. In the observation group (43 cases), there were 24 males and 19 females, with an average age of (70.54±8.14) years old (62~81 years old) and an average disease course of (11.22±5.79) years; there were 10 cases of cardiac functional grade II, 27 cases of grade III and 6 cases of grade IV. The differences of general data between the two groups had no statistical significance (*P*>0.05).

### Comparison of blood gas analysis results between two groups

The differences of pH, PaO_2_ and PaCO_2_ between the two groups had no statistical significance before treatment (P>0.05). After treatment, the blood gas analysis indexes in both groups significantly improved, and the differences were statistically significant (P<0.05); the improvement of the indexes in the observation group was superior to that of the control group (P<0.05) (Table-I).

**Table-I:** Comparison of blood gas analysis indexes between the two groups.

	*Group*	*Observation group*	*Control group*	*T*	*P*
pH	Before treatment	7.22±0.12	7.21±0.09	1.167	> 0.05
	After treatment	7.43±0.03	7.35±0.08	0.978	> 0.05
	t	9.875	6.742		
	P	<0.05	<0.05		
PaO2 (mmHg)	Before treatment	40.23±3.12	40.45±3.03	0.714	>0.05
	After treatment	68.13±3.03	60. 01±4.72	7.137	<0.05
	t	10.053	9.609		
	P	<0.05	<0.05		
PaCO2 (mmHg)	Before treatment	57.32±5.51	57.45±5.74	2.464	>0.05
	After treatment	45.08±2.27	38.93±1.61	11.724	<0.05
	t	6.530	4.292		
	P	< 0.05	< 0.05		

### Comparison of lung function and 6-minutes walking distance between the two groups

The differences of FEV_1_, FVC and 6-minutes walking distance between the two groups had no statistical significance before treatment (*P*<0.05). The FEV_1_, FVC and 6-minutes walking distance of both groups after treatment were higher than those before treatment; the improvement of the observation group was more obvious, and the difference had statistical significance (*P*<0.05) (Table-II).

**Table-II:** Comparison of FEV1, FVC and 6-min walking distance between the two groups.

	*Group*	*Observation group*	*Control group*	*t*	*P*
FEV1 (L)	Before treatment	0.66±0.10	0.68±0.09	0.816	>0.05
	After treatment	1.08±0.11	0.93±0.09	10.367	>0.05
	t	10.416	8.430		
	P	<0.05	<0.05		
FVC (L)	Before treatment	1.42±0.31	1.45±0.43	0.769	>0.05
	After treatment	2.1±0.2	1.7±0.1	6.879	<0.05
	t	14.573	10.245		
	P	<0.05	<0.05		
6-min walking distance (m)	Before treatment	251.28±34.21	257.63±50.38	1.245	>0.05
	After treatment	380.34±31.28	302.79±29.74	9.887	<0.05
	t	64.556	34.138		
	P	<0.05	<0.05		

### Comparison of pulmonary arterial pressure between the two groups

The peripheral systolic blood pressure (PSBP) and peripheral diastolic blood pressure (PDBP) of the two groups decreased after treatment compared to those before treatment; the decrease amplitude of the observation group was larger than that of the control group (p < 0.05) (Table-III).

**Table-III:** Comparison of PSBP and PDBP between the two groups (mmHg, mean±SD).

*Group*	*Time point*	*PSBP*	*PDBP*
Observation group	Before treatment	36.74±0.73	28.32±0.45
	After treatment	35.43±0.56*	25.67±0.68*
Control group	Before treatment	36.61±0.84	28.22±0.55
	After treatment	36.16±0.51	26.97±0.84

***Note:*** PSBP: peripheral systolic blood pressure, PDBP: peripheral diastolic blood pressure; HDL-C: high density lipoprotein cholesterol;

* indicated P <0.05, compared to the control group.

## DISCUSSION

COPD featured by incomplete reversible airway limitation and increased inflammatory response can severely threaten the physical and mental health of people. A study suggested that, the incidence of COPD among people who are over 40 years old can be 8.2% in China and about 6% of the cases may develop to chronic pulmonary heart disease.^10^ Currently, conventional symptomatic treatment is the major therapy for COPD in combination with pulmonary arterial hypertension; it relieves the clinical symptoms through measures such as assisted oxygen inhalation, cardiac reinforcement and dieresis, but its effect is not quite ideal.^11^ The occurrence basis of COPD in combination with pulmonary arterial hypertension is in a close correlation with pulmonary artery vasoconstriction, vascular structure abnormality and endovascular blood flow resistance; hence the treatment for COPD in combination with pulmonary arterial hypertension should focus on the reconstruction of vascular structure and the reduction of endovascular blood flow resistance.

Macrolides with a strong antioxidation can reduce the damages induced by increased neutrophil. Azithromycin as a representative of macrolides can effectively inhibit the level of interleukin-6. Azithromycin can effectively restrain the adhesion of neutrophil to epithelial tissue, reduce the secretion of sputum, and inhibit inflammatory reaction by inducing the apoptosis of macrophage and neutrophil through immunoregulation. Because of the effects mentioned above, macrolides can significantly improve the lung function of COPD patients.^12^ Simvastatin which was used for lowering blood lipid previously was found with a favorable effect on vascular reconstruction and right ventricular hypertrophy of patients with pulmonary arterial hypertension in recent years. It can promote the generation of endothelial cells, relieve and recover endothelial function, relieve inflammatory effect, inhibit the expression of endothelin (ET) and angiotensin, block the generation of vascular smooth muscle cells, weaken the activity of matrix metalloproteinase, restrain platelet aggregation, and regulate blood viscosity. It improves and recovers endothelial cell function by lowering the transcription rate of ET-1, inhibiting vasoconstriction, reduce the expression of vascular endothelial cell growth factor, block the generation of reactive oxygen molecules, and controlling the mitosis and proliferation of vascular endothelial cells, thus to effectively reduce pulmonary arterial hypertension.^13^,^14^

COPD in combination with pulmonary arterial hypertension has been attached more and more importance by the respiratory field. The research results obtained by scholars in China and abroad are constantly updated. Noteboom B et al. found that,^15^ azithromycin had a remarkable clinical effect on COPD as it could improve lung function by significantly increasing 6-minutes walking distance and lowering 24-hour sputum quantity and dyspnea scores. Through treating COPD in combination with pulmonary arterial hypertension with roxithromycin and simvastatin, Pang Y et al. found that,^16^ FEV_1_% and St George’s respiratory questionnaire score were improved after treatment and the improvement of patients who were treated by roxithromycin in combination with simvastatin was superior to those who were treated by roxithromycin or simvastatin. The results of this study suggested that, the blood gas analysis indexes of the observation group had a higher improvement compared to those of the control group; there were remarkable differences in FEV_1_, FVC and 6-minutes walking distance between the observation group and the control group, and the PSBP and PDBP of the observation group were lower than those of the control group, suggesting azithromycin in combination with simvastatin could dramatically regulate pulmonary arterial pressure and improving lung function.

## CONCLUSION

Treating COPD in combination with pulmonary arterial hypertension is safe and reliable. The therapy can remarkably relieve ventilation dysfunction and provide a theoretical basis for the application of safe and effective drugs in the treatment of COPD in combination with pulmonary arterial hypertension. Due to the small sample size and short research time, large-scale, random and multi-center clinical trials are needed for further verification.

### Authors’ Contribution

**PDW:** Conceived the study.

**JY & YWY:** Prepared the first draft.

**PDW & ZXD:** Collected, analyzed the data and revised the paper.
